# Endoscopic removal of a transgastric migrated textiloma: a case report

**DOI:** 10.2144/fsoa-2022-0059

**Published:** 2023-03-13

**Authors:** Ghada Gharbi, Sondes Bizid, Ghanem Mohamed, Riadh Bouali

**Affiliations:** 1 Department of Gastroenterology, Principal Military Hospital of Instruction of Tunis, Tunis, Tunisia

**Keywords:** abdominal textiloma, endoscopic removal

## Abstract

**Aim:**

Abdominal textiloma is an uncommon postoperative complication which can result in a fistula with luminal migration in the digestive tract. Surgery has been the mainstay method for textiloma removal; however, removal of retained gauze by upper gastrointestinal endoscopy is possible avoiding reoperation.

**Case report:**

We report a case of an abdominal textiloma in a 38-year-old male, which migrated into the stomach and was extracted by upper endoscopy.

**Conclusion:**

Endoscopic extraction after a luminal migration of the abdominal textiloma in the digestive tract facilitate its management and could avoid surgery.

## Plain language summary

The diagnosis of abdominal textiloma must be considered in front of unexplained symptoms in patients with a surgical history. Surgery has been the mainstay method for textiloma removal; however, endoscopic removal may be the best option which avoids reoperation. We report a case of an abdominal textiloma in a 38-year-old male, which migrated into the stomach and was extracted by upper endoscopy.

Abdominal textiloma, also called gossypiboma is a rare postoperative complication caused by gauze fibers retained during surgery [1]. Retained surgical foreign bodies occur one out of every 1000–1500 abdominal cavity operations [2]. Transmural migration of retained surgical swab is also reported [2]. It is an uncommon complication, in which the endoscopic treatment could avoid reoperation.

We report a case of an abdominal textiloma in a 38-year-old male, which migrated into the stomach and was extracted by upper endoscopy.

## Case presentation

A 38-year-old male, with a history of sleeve gastrectomy, consulted 1 month later for dysphagia and an infectious syndrome. The abdominal examination was normal and the laboratory tests showed an inflammatory biological syndrome (white blood cells = 11,560/mm^3^, CRP = 113 mg/ml).

**Figure 1.**
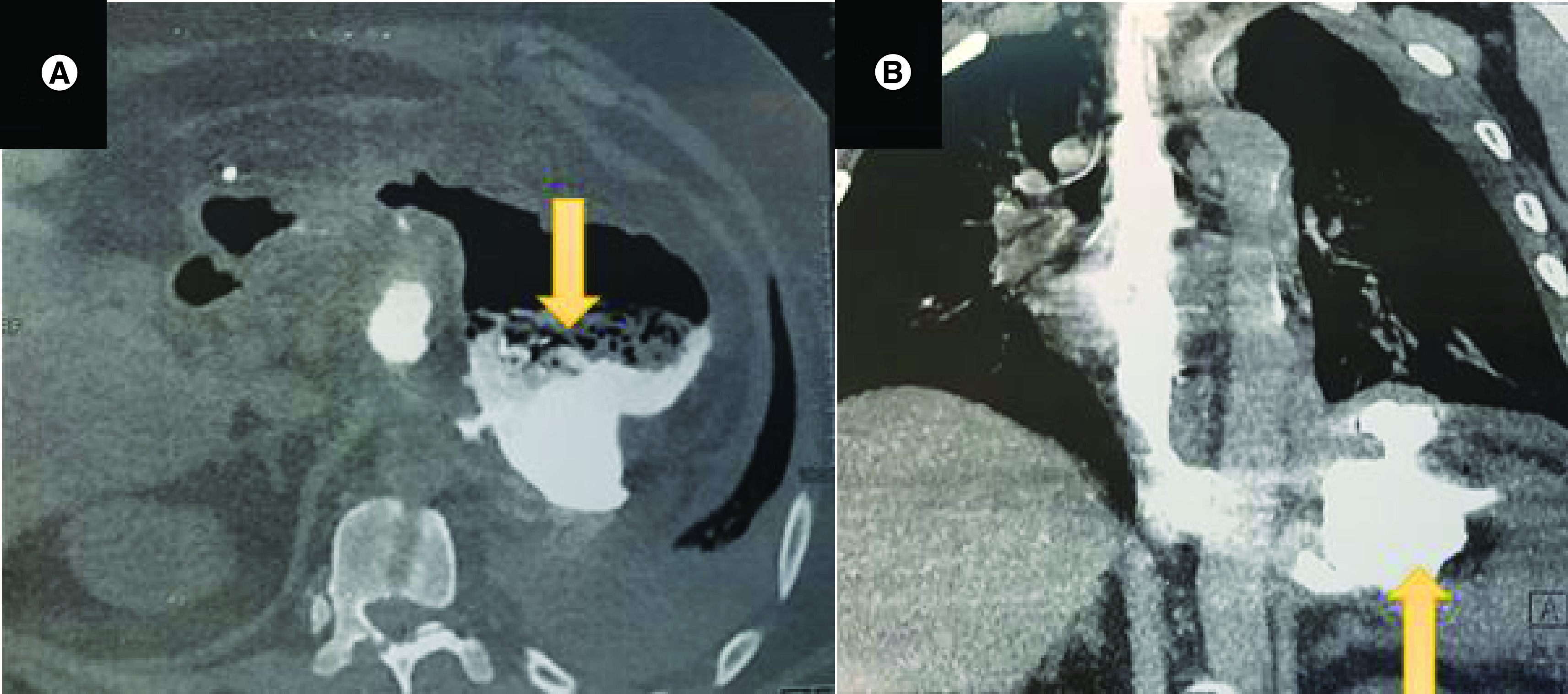
(A) CT scan axial view showing a left subphrenic collection (yellow arrow) communicating with the gastric sleeve. **(B)** CT scan coronal view showing the left subphrenic collection (yellow arrow).

**Figure 2.**
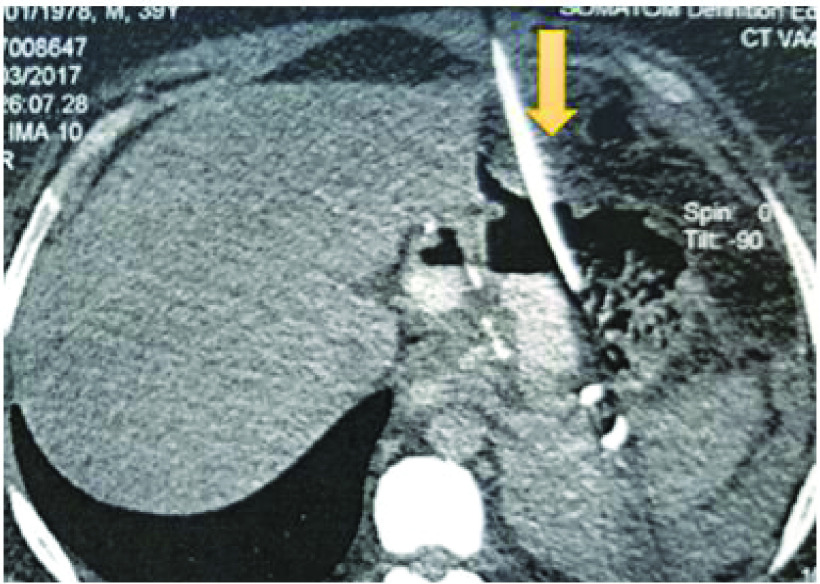
A coronal abdominal CT section showing the percutaneous radiological drainage (yellow arrow).

Computed tomography (CT) of the abdomen showed a left subphrenic collection communicating with the gastric sleeve (Figure 1). A percutaneous radiological drainage was performed (Figure 2) and complicated with an early leakage at the upper pole of the stapling line. The abdominal CT revealed a 12 mm hole with a 7 cm perigastric collection and without an associated stenosis of the gastric sleeve.

**Figure 3.**
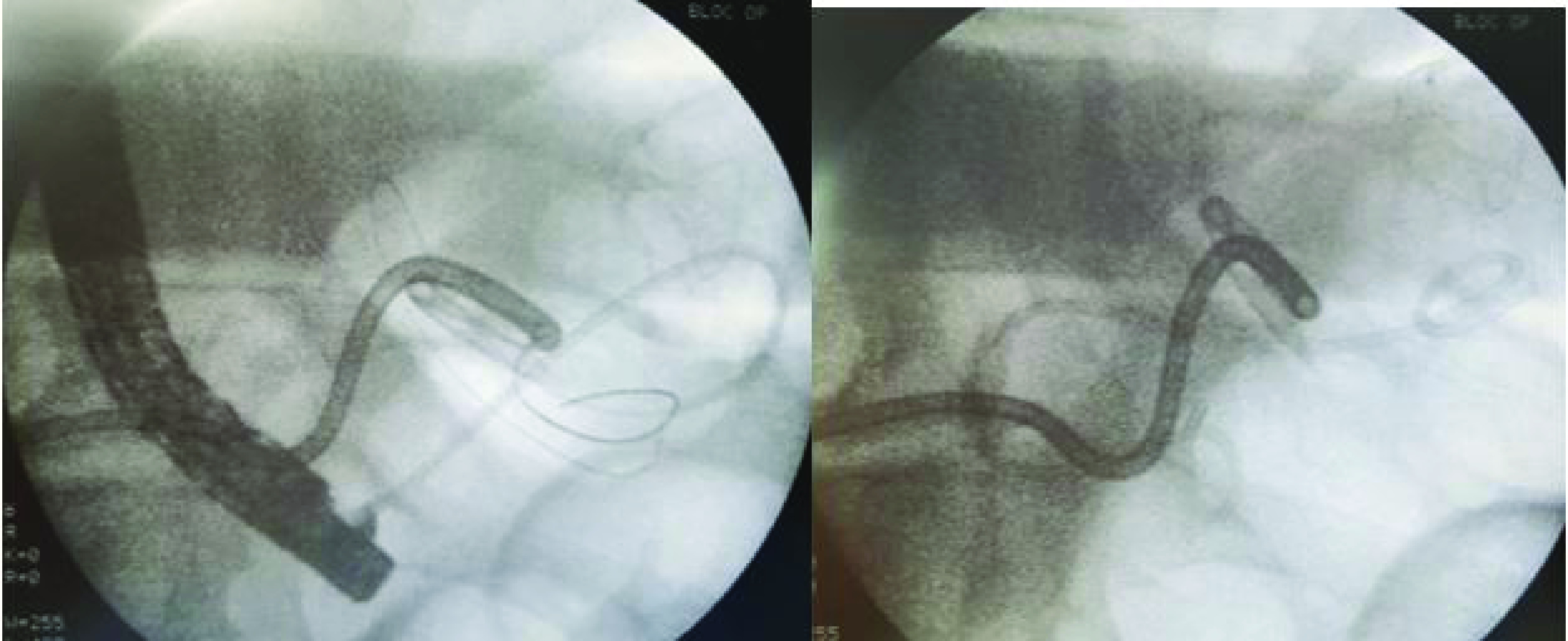
X-ray showing the endoscopic internal drainage with double pigtail plastic stent.

Endoscopic internal drainage with double pigtail plastic stent was performed (Figure 3).

**Figure 4.**
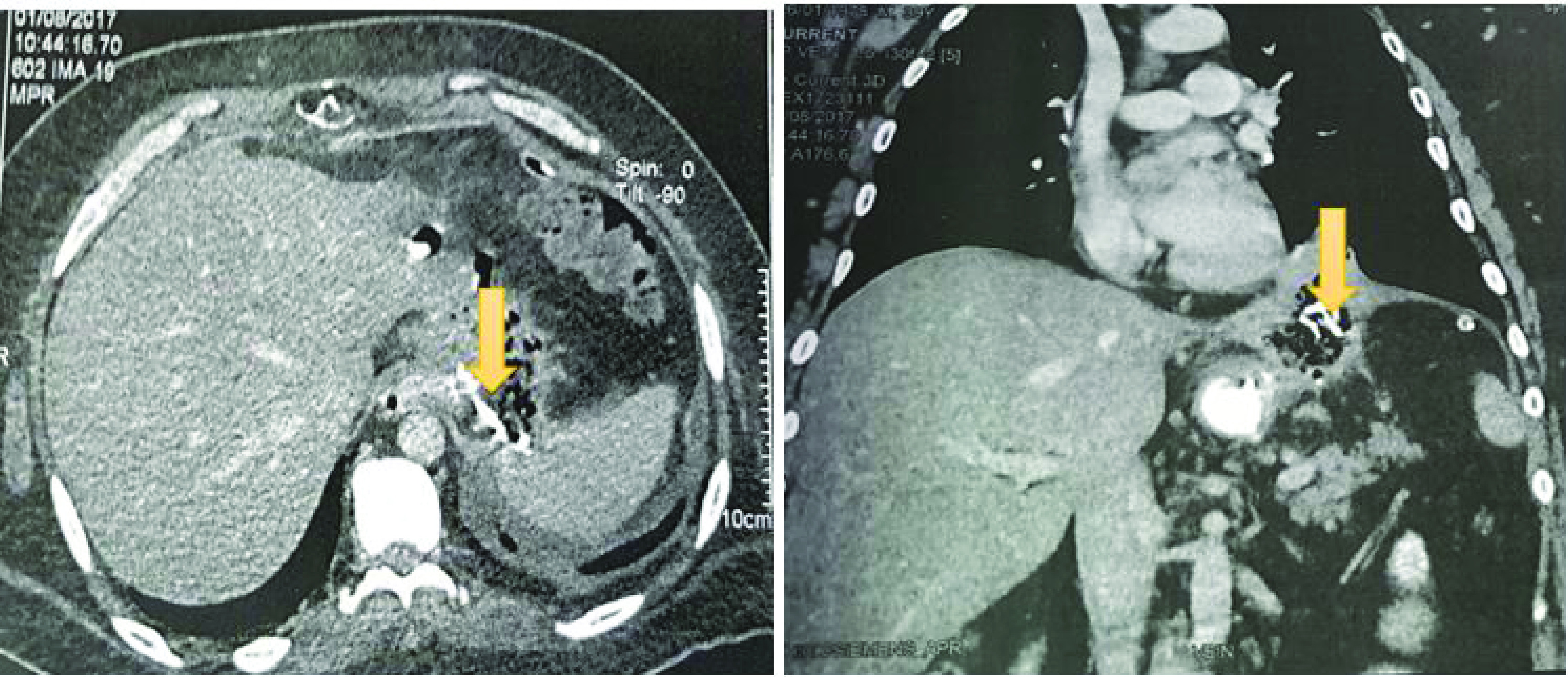
CT scan axial view showing a slight decrease in the size of the subphrenic collection with a persistent fistula and a spongiform formation with opaque serpiginous structures (yellow arrow) typical of a textiloma.

The CT evaluation 5 months later found a slight decrease in the size of the subphrenic collection with a persistent fistula and a spongiform formation with opaque serpiginous structures typical of a textiloma (Figure 4).

**Figure 5.**
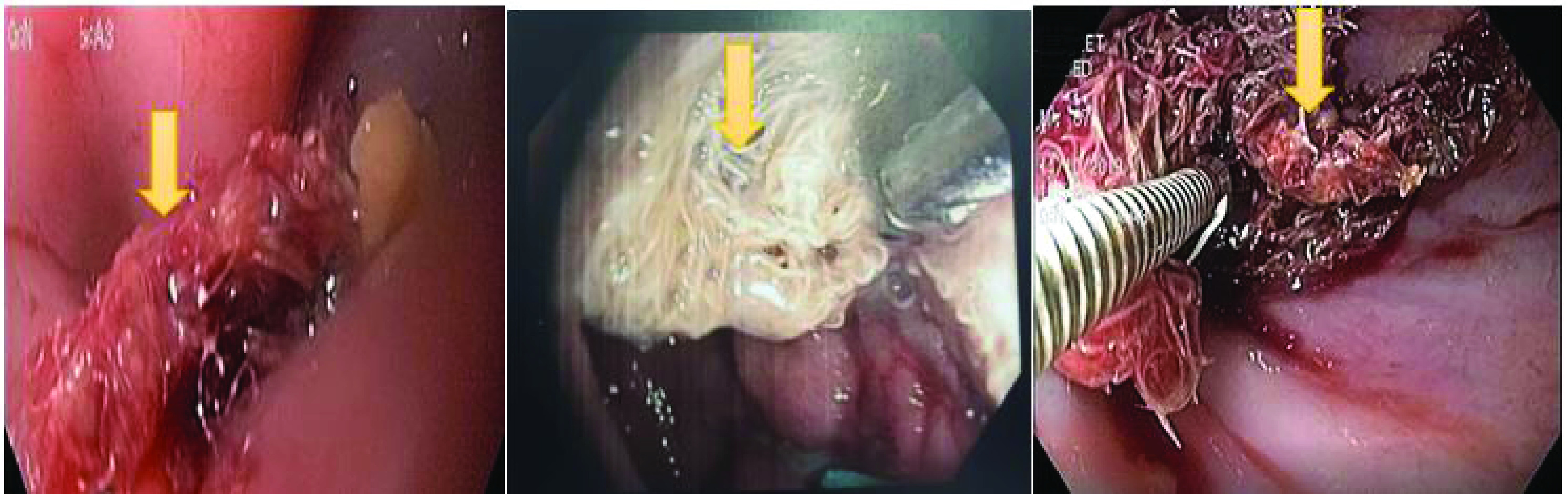
Upper endoscopy showing a narrowed cardia with a 10 mm loss of substance on the right edge blocked by a textiloma (yellow arrow).

The upper endoscopy showed a narrowed cardia with a 10 mm loss of substance on the right edge blocked by a textiloma (Figure 5).

Endoscopic extraction of the entire textiloma with the foreign body forceps in several fragments was successfully made.

Patient reported a relief of the previous dysphagia and the abdominal CT 1 month later showed a clear regression of the perigastric cavity. Unfortunately, CT images after endoscopic removal of textiloma were not available.

## Discussion

Abdominal textiloma is an iatrogenic postoperative complication resulting in textile fibers forgotten in the abdominal cavity during surgery.

It has become very rare as safety rules in the operating room follow precise guidelines [3]. However, it must be suspected in front of pain, abdominal mass or signs of infection in patients with a history of abdominal surgery [4]. The time to diagnosis of gossypiboma varies from few days to many years after surgery. It depends on the type of response elicited by the fibers [5].

In fact, this foreign body could lead either to an exudative inflammatory or an aseptic fibrotic reaction encapsulating the material and leading to a mass [5].

It could then lead to intra-abdominal abscess formation, like our patient in whom the forgotten compress result in a subphrenic abscess, or in a mass which could simulate a tumor [3].

A necrosis of the intestinal wall can occur as a result of the pressure and irritation on the digestive tract. A Fistula occurs when the foreign body erodes into the lumen [5].

Risk factors associated with increased incidence of retained surgical foreign bodies are complex surgical procedures, open abdominal procedures, staged and abbreviated procedures, emergency surgical procedures, increasing BMI, participation of more than one surgical team, procedures involving many body cavities, change in surgical procedure, use of many instruments [6].

The clinical presentation is unspecific depending on the exact site where the retained gauze is located [7]. It can remain asymptomatic. In the case of migration of the item into the lumen of the stomach, small intestine or colon, the patient would present nausea and vomiting. The multiple intestinal fistulas or intraluminal bacterial overgrowth lead into a malabsorption syndrome with weight loss [8]. Bowel obstruction can also complicate gossypiboma. It can concern either the small intestine [4,7,9] or the colon [8].

In our patient, the compress migrated into the stomach and blocked in the cardial region causing dysphagia.

Diagnosis is difficult to establish as it can mimic hematoma, abscess, granulomatous process or even a cystic mass or a neoplasm [10]. If a radio-opaque marker has been impregnated in the foreign body, abdominal radiograph can help the diagnosis [11]. It is also useful in case of intestinal obstruction or bowel perforation. On ultrasonography, a gossypiboma is usually visualized as a well-defined mass with wavy internal echogenic structures showing posterior acoustic shadowing [12].

The CT scan is the modality of choice to diagnose abdominal textiloma. The pathognomonic characteristic feature is spongiform or mottled pattern due to air bubbling [11]. Other suggestive feature is a well-defined mass with a dense enhanced capsule and variable density and calcification [13].

The typical feature on MRI is a soft-tissue mass with a thick well-defined capsule on T2-weighted imaging and peripheral wall enhancement and central stripes gadolinium injection [1,14].

As far as treatment is concerned, open surgery has been the mainstay method for textiloma removal [15]. Few cases have been reported in relation with removal of retained gauze by upper gastrointestinal endoscopy. The first case was reported by Sozutek *et al.*, where a 20 cm × 20 cm surgical sponge was endoscopically removed [16]. In our patient, textiloma was successfully removed by upper endoscopy in several fragments. The most used tool for endoscopic removal is endoscopic forceps with sometimes the need of a saw-tooth forceps [15,16].

Prognosis is excellent if abdominal textiloma is removed early after diagnosis. However, when diagnosis and treatment are dalayed, mortality rates varies between 10 and 17% [8].

Despite sponge count during the operative checklist, foreign bodies can be forgotten in the abdominal cavity.

News tools as bar codes automated counting systems and counters, radiofrequency labelled sponges and radio frequency readers can avoid manual errors in counting [17,18].

Indeed, the use of sponges impregnated with radio-opaque substances with an intraoperative abdominal radiograph just before the closure can help detecting a foreign body [19].

An excellent communication between the surgical team, operating room nurses and anesthetists is mandatory to reduce the incidence of abdominal textiloma.

## Conclusion

Abdominal textiloma is an avoidable postoperative complication which can have devastating consequences on the patient and medicolegal implications of the surgeon. Endoscopic extraction after a luminal migration of the foreign body in the digestive tract facilitate its management and could avoid a second surgery. However, prevention remains the best tool.

**Summary points**

- The diagnosis of abdominal textiloma must be considered in front of an unexplained symptoms in patient with surgical history.
- Endoscopic extraction after a luminal migration of the abdominal textiloma in the digestive tract facilitate its management and could avoid surgery.
- Prevention of abdominal textiloma remains the best tool to avoid complications.
